# Insight from convergent validity of assessment of systemic sclerosis–associated Raynaud phenomenon with nailfold capillaroscopy

**DOI:** 10.1093/rap/rkaf122

**Published:** 2025-10-21

**Authors:** Krishnasai Abhishek Madathanapalli, Areeka Memon, Manvitha Nadella, Kyra Shelton, Michael Zamani, Amrita Makhijani, Alyssa Williams, Ilayda Gunes, Sophia Esme Kujawski, William Odell, Stephanie Perez, Nicolas Page, Lucy Duran Camacho, Cassandra Van Horn, Francis Perry Wilson, Robyn Domsic, Lan Yu, John D Pauling, Monique Hinchcliff

**Affiliations:** Section of Rheumatology, Allergy & Immunology, Yale School of Medicine, New Haven, CT, USA; Section of Rheumatology, Allergy & Immunology, Yale School of Medicine, New Haven, CT, USA; Section of Rheumatology, Allergy & Immunology, Yale School of Medicine, New Haven, CT, USA; Department of Internal Medicine, Section of Nephrology, Yale School of Medicine, New Haven, CT, USA; Independent Statistician, Washington, DC, USA; Department of Internal Medicine, Section of Nephrology, Yale School of Medicine, New Haven, CT, USA; Section of Rheumatology, Allergy & Immunology, Yale School of Medicine, New Haven, CT, USA; Section of Rheumatology, Allergy & Immunology, Yale School of Medicine, New Haven, CT, USA; Section of Rheumatology, Allergy & Immunology, Yale School of Medicine, New Haven, CT, USA; Section of Rheumatology, Allergy & Immunology, Yale School of Medicine, New Haven, CT, USA; Section of Rheumatology, Allergy & Immunology, Yale School of Medicine, New Haven, CT, USA; Section of Rheumatology, Allergy & Immunology, Yale School of Medicine, New Haven, CT, USA; Section of Rheumatology, Allergy & Immunology, Yale School of Medicine, New Haven, CT, USA; Section of Rheumatology, Allergy & Immunology, Yale School of Medicine, New Haven, CT, USA; Department of Internal Medicine, Section of Nephrology, Yale School of Medicine, New Haven, CT, USA; Department of Internal Medicine Clinical and Translational Research Accelerator, Yale School of Medicine, New Haven, CT, USA; Department of Medicine, University of Pittsburgh, Pittsburgh, PA, USA; Department of Medicine, University of Pittsburgh, Pittsburgh, PA, USA; Department of Rheumatology, North Bristol NHS Trust, Bristol, UK; School of Translational Health Sciences, Bristol Medical School, University of Bristol, Bristol, UK; Section of Rheumatology, Allergy & Immunology, Yale School of Medicine, New Haven, CT, USA; Department of Internal Medicine Clinical and Translational Research Accelerator, Yale School of Medicine, New Haven, CT, USA

**Keywords:** SSc, scleroderma, Raynaud phenomenon, digital nailfold capillaroscopy, health-related quality-of-life, assessment of systemic sclerosis-associated Raynaud phenomenon (ASRAP), Dinolite, outcome measures, digital ulcers, patient-reported outcome measures

## Abstract

**Objectives:**

The assessment of systemic sclerosis–associated Raynaud phenomenon (ASRAP) questionnaire was recently developed. We tested the convergent validity of the ASRAP instrument with patient demographics and capillary morphology on nailfold capillaroscopy (NFC).

**Methods:**

Participants completed the ASRAP (range 20–80; higher scores unfavourable) and underwent NFC using Dinolite™. We collected demographic and relevant clinical data. Two blinded assessors classified NFC images as ‘scleroderma pattern’ present or absent and recorded specific abnormalities. We assigned the NFC semi-quantitative score (range 0–3) based on the frequency of these abnormalities. Descriptive statistics were generated, and regression and correlation analyses were used to evaluate associations between ASRAP, NFC and clinical factors.

**Results:**

Seventy-five patients [87% women, 78% white, 77% with limited cutaneous SSc] with a mean (SD) age of 58 (14) were recruited from scleroderma clinics. ASRAP scores were 11.2 points higher in women compared with men with other significant variables held constant. The NFC semi-quantitative score weakly correlated with ASRAP (Spearman’s σ = 0.23, *P* = 0.05), with moderate correlation in winter (Spearman’s σ = 0.36, *P* = 0.04). Holding other variables constant, patients with dilated capillaries and capillary dropout had higher ASRAP scores by 6.1 and 6.8 points, respectively.

**Conclusion:**

In SSc patients, females reported higher ASRAP scores. Greater proportion of ‘scleroderma pattern’ NFC abnormalities as well as specific abnormalities like dilated capillaries and capillary dropout were associated with higher ASRAP providing mechanistic insight and supporting the construct validity of ASRAP for assessing SSc-RP.

Key messages

- ASRAP scores are higher in people with more advanced obliterative microangiopathy in SSc.
- Female gender is associated with higher ASRAP questionnaire scores.
- Our results support the construct validity of the ASRAP questionnaire for measurement of SSc-associated Raynaud phenomenon.

## Introduction

Raynaud phenomenon (RP) is a microvascular vasospastic disorder characterized by visible skin colour changes triggered by cold, stress and/or environmental factors reported by ≥95% of patients with systemic sclerosis (SSc) [1]. In addition to vasospasm, people with SSc manifest an obliterative microangiopathy which can result in severe RP symptoms and ischaemic tissue injury including digital ulcerations (DU) [2]. Morphological capillary abnormalities can be directly visualized in SSc using nailfold capillaroscopy (NFC) [3]. Demonstrating the efficacy of promising pharmacotherapeutics for SSc-RP has been challenging using traditional diary-based patient-reported outcome (PRO) approaches for assessing SSc-RP attack characteristics [4]. RP attack diaries do not take into consideration the significant adaptation of patients to avoid or ameliorate RP symptoms [5, 6].

The assessment of systemic sclerosis–associated Raynaud phenomenon (ASRAP) questionnaire is a new patient-reported outcome measure (PROM) developed by patient research partners, psychometricians and a team of SSc-RP experts [7]. The ASRAP includes items that measure the burden of RP across different physical and emotional domains and was developed to overcome limitations of existing RP PROMs. The development and validation of ASRAP is described in full elsewhere [8]. In brief, the ASRAP questionnaire items were devised to capture the experiences of RP in SSc with extensive patient input including cognitive debriefing and linguistic testing. The scoring and calibration of ASRAP was developed following extensive structural validity testing including exploratory/confirmatory factor analysis and assessment of internal consistence. In the largest prospective study of SSc-RP undertaken to date, we confirmed excellent repeatability and strong construct validity on psychometric testing as well as excellent discriminant validity with higher ASRAP scores in patients with DUs [9]. SSc-DU are a major cause of disease-related morbidity in SSc and occur in patients with extensive capillary dropout on NFC [10].

The importance of NFC has grown since abnormal NFC was included in the ACR/EULAR 2013 SSc classification criteria as well as the Very Early Diagnosis of Systemic Sclerosis (VEDOSS) criteria [11]. The Dinolite™ AF4515-N2UT Digital USB Microscope (Dinolite) offers a convenient, handheld and relatively inexpensive method for NFC comparable to the gold standard videocapillaroscopy [12]. No previous studies have examined the relationship between the severity of obliterative microangiopathy and the impact of SSc-RP assessed using ASRAP. Herein, we undertook a cross-sectional study to assess the convergent validity of the ASRAP in an independent US cohort by comparing scores to digital NFC and other clinical features.

## Methods

### Study participants and design

The Yale Human Research Protection Program Institutional Review Board (IRB) (#2000026608) approved the study of patients who fulfilled ACR/EULAR 2013 SSc classification criteria. Following informed consent, patients with self-reported active RP were consecutively and prospectively recruited from December 2021 through November 2023. Participants completed the ASRAP electronically using tablet computers and underwent Dinolite NFC.

### Demographic and clinical data collection

We obtained demographic data including age, patient-reported gender, race and ethnicity. We collected clinical data: SSc subtype (limited cutaneous, diffuse cutaneous, SSc sine scleroderma and mixed connective tissue disease), ANA positivity and pattern, SSc specific antibody positivity [anticentromere, anti-topoisomerase I (Scl-70) and RNA polymerase III (RNA pol III)], duration from RP onset to study entry, modified Rodnan skin score (mRSS), history of DU (defined as an area of visually discernible depth and a loss of continuity of epithelial coverage, which can be denuded or covered by necrotic tissue and/or a scab) [13], presence of pulmonary arterial hypertension (PAH) defined as mean pulmonary artery pressure (mPAP) greater than 20 mm Hg together with a normal pulmonary artery wedge pressure (less than or equal to 15 mm Hg) on right heart catheterization [14], active medications and cigarette smoking status (never, previous, active).

### Nailfold capillaroscopy

Following 30-min acclimatization at constant ambient temperature and application of immersion oil, we performed NFC using Dinolite’s polarizing mode. Four fingers per hand (four central nail fold images per finger at 180–220 X magnification), excluding the thumb, were imaged [3]. Two trained and blinded assessors classified NFC images as having or lacking the ‘scleroderma pattern’ based on the presence of characteristic abnormalities in capillary density, dimension, morphology and presence of hemorrhages [3]. Additionally, the presence or absence of specific NFC abnormalities were recorded including tortuous, dilated, meandering, irregularly enlarged and bizarre capillaries, neoangiogenesis, microhemorrhages, capillary dropout and avascular areas [15]. Supplementary Table S1, available at *Rheumatology* Online, and Supplementary Fig. S1, available at *Rheumatology* Online, define and illustrate these variables. We assigned a semi-quantitative score by rating the distal nailfold capillary row as 0 = no changes, 1 = ≤ 33% of capillary alterations/reduction, 2 = 34–66% of capillary alterations/reduction and 3 = ≥ 67% of capillary alterations/reduction consistent with the ‘scleroderma pattern’ [16].

### Patient-reported outcome measure

Participants completed the ASRAP questionnaire electronically with responses directly entered into a study specific Research Electronic Data Capture (RedCap™) (v 14.5.43) database. The ASRAP includes 27 questions that are categorized into the following domains: physical symptoms of RP, frequency and duration of RP attacks, emotional impact, impact on daily life, impact of cold, management and uncertainty due to RP symptoms. ASRAP T-scores were calculated using the item parameter estimates from the item response theory (IRT) calibration of the ASRAP full bank. The ASRAP T-scores have a mean of 50, and SD of 10. Scores range from 20 to 80 with higher scores indicating worse RP severity and impact [9].

### Statistical analysis

We tested the normality of data distribution using the Shapiro–Wilk test. We presented descriptive statistics as median (IQR) for non-normal continuous variables, mean (SD) for normal continuous variables and numbers (percentages) for discrete variables. Continuous variable statistics were compared via Kruskal–Wallis test contingent on the number of groups, while the Fisher’s exact test compared categorical variables. We used descriptive statistics to assess ASRAP and mean semi-quantitative score distributions across seasons (spring: 3/1–5/31; summer: 6/1–8/31; fall: 9/1–11/30; winter: 12/1–2/28). Spearman’s rank test evaluated the association between ASRAP and mean semi-quantitative scores by season. We used the rank sum test to compare ASRAP T-scores between participants with and without specific NFC abnormalities. Linear regression estimated significant impacts of NFC abnormalities on ASRAP score controlling for patient characteristics (age, gender, race, time since onset of RP, season of measurement, history of DUs). Inter-rater reliability was assessed using Cohen’s kappa for abnormality detection and semi-quantitative score across the same set of raters. A pooled kappa statistic was then calculated by taking the weighted average of the individual kappa statistics to summarize overall agreement across the two items. Spearman’s rho <0.3 was considered weak, 0.3–0.7 moderate and >0.7 strong correlation [17]. Given the rarity of missing observations, and the absence of a relationship between missingness and observation of other variables, we assumed the mechanism to be missing completely at random (MCAR). All analyses were performed using R version 4.4.2.

## Results

### Baseline characteristics

Seventy-five SSc-RP participants with a mean (SD) age of 58 (14) years were consecutively recruited (Table 1). The patient cohort was 87% female, 78% white and 9% Hispanic. The disease subtypes included limited cutaneous (77%), diffuse cutaneous (15%), SSc sine scleroderma (7%) and mixed connective tissue disease (1%). Twelve patients (16%) had a history of DU, 10 (13%) had PAH and 47 (62%) were receiving vasodilatory RP treatment. The median (IQR) time since RP diagnosis was 10 (5–22) years, and the median (IQR) mRSS was 4 [2–6]. Positive ANA was found in 87%, anticentromere in 48%, anti-Scl-70 in 19% and anti-RNA Pol III in 11%. The mean (SD) ASRAP score was 44.6 (10.5). Participants with missing data—two without RP onset date, two without ANA, one without Scl-70, two without anticentromere and one without RNA Pol III—were omitted from analyses.

**Table 1.** Baseline characteristics of SSc-RP cases

Patient characteristic	SSc-RP cases (*N* = 75)
Age (years), mean (SD)	57.72 (13.91)
Gender, *n* (%)	
Male	10 (13%)
Female	65 (87%)
Race, *n* (%)	
Black	6 (8%)
Asian	0 (0%)
White	58 (77.33%)
Other^a^	4 (5%)
Hispanic ethnicity, *n* (%)	7 (9%)
SSc disease subtype *n* (%)	
LcSSc	58 (77%)
DcSSc	11 (15%)
SSc sine scleroderma	5 (7%)
Mixed connetective tissue disease (MCTD)	1 (1%)
History of digital ulcers, *n* (%)	12 (16%)
Diagnosis of pulmonary hypertension, *n* (%)	10 (13.33%)
Receiving RP treatment, *n* (%)	47 (62%)
Time since RP diagnosis, median (IQR)	10 (5-22)
Modified Rodnan skin score, median (IQR)	4 (2-6)
Positive ANA, *n* (%)	65 (87%)
SSc specific serum antibodies	
Anticentromere	36 (48%)
Anti-Scl-70	14 (19%)
Anti-RNA polymerase III	8 (11%)
ASRAP score, mean (SD)	44.59 (10.54)
NFC semi-quantitative score, *n* (%)	
0: No changes	12 (16.0%)
1: ≤ 33% of capillary alterations/reduction	21 (28.0%)
2: 34–66% of capillary alterations/reduction	16 (21.3%)
3: ≥ 67% of capillary alterations/reduction	26 (34.7%)

a Other includes participants who identified as Alaskan Native or American Indian, Middle Eastern or OTHER. ASRAP: assessment of systemic sclerosis–associated Raynaud phenomenon; RP: Raynaud phenomenon.

### Patient characteristics and ASRAP

Significant associations between age, gender, race/ethnicity, DU history, season and ASRAP scores were determined using univariate linear regression (Supplementary Table S2, available at *Rheumatology* Online). Here, we found mean ASRAP scores were 12 points higher for women than men, 11.3 points higher for Hispanics than non-Hispanics, and 7.9 points higher for those with history of DU compared with those without. After controlling for significant factors identified in univariate models, mean ASRAP scores for women were 11.2 points higher than men (Supplementary Table S3, available at *Rheumatology* Online), while there were no significant differences in ASRAP scores based on Hispanic ethnicity and DU history.

### NC abnormalities and ASRAP

**Figure 1.**
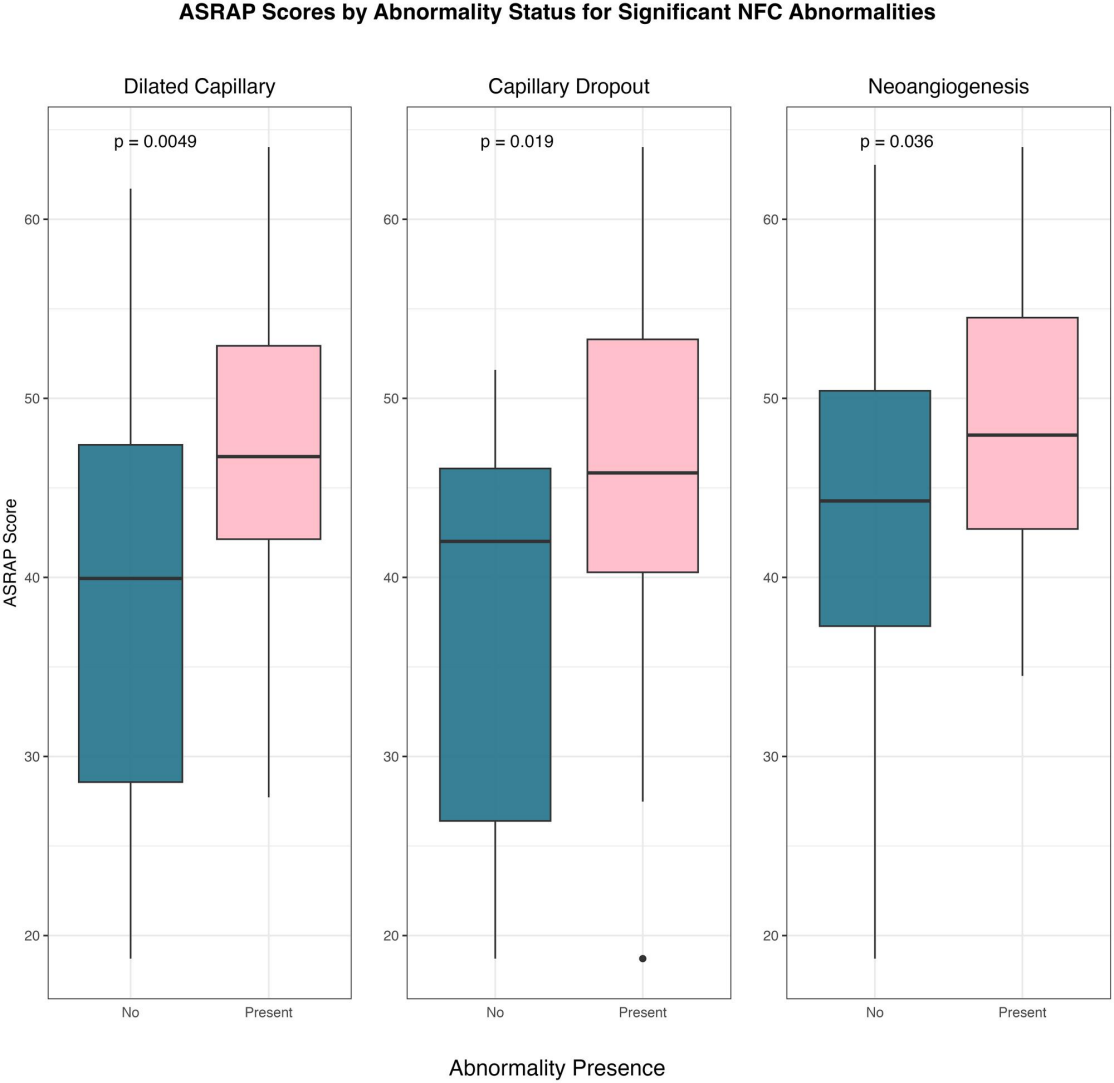
Boxplot comparing ASRAP scores of patients with and without specific NFC abnormalities. ASRAP: assessment of systemic sclerosis–associated Raynaud phenomenon

We found a weak correlation between semi-quantitative NFC score and ASRAP (σ = 0.23, *P* = 0.05) which improved to moderate correlation when limiting our analysis to data obtained in cold weather months (σ = 0.36, *P* = 0.04) to negate the impact of seasonal variation in weather. Patients with dilated capillaries, neoangiogenesis and capillary dropout (but not other abnormalities) had higher ASRAP scores than those without [mean (SD)] [47.3 (8) vs 39.2 (12.9) *P* = 0.004, 48.9 (7.7) vs 43 (11) *P* = 0.03, 46.1 (9.7) vs 37.2 (11.7) *P* = 0.02, respectively] (Fig. 1). On average, patients with NFC semi-quantitative score of 3 had higher ASRAP scores compared with those with a score = 0 (β  =  9.7) (Supplementary Table S2, available at *Rheumatology* Online). Controlling for variables that showed a significant correlation with ASRAP in univariate analyses, patients with any dilated capillaries and capillary dropout had higher ASRAP scores than those without (β  =  6.1 and 6.8, respectively) (Supplementary Table S3, available at *Rheumatology* Online). There was substantial inter-rater agreeability between our two blinded NFC assessments with a pooled kappa value of 0.67 (95% CI: 0.51–0.84).

## Discussion

RP is common in patients with SSc and causes decreased health-related quality of life (HRQoL). Equivocal results from RP clinical trials raise questions about SSc-RP outcomes including PROMs [6]. The novel ASRAP questionnaire assesses domains that patients living with SSc-RP have identified as important beyond frequency and duration of RP ‘attacks’ measured by legacy instruments [7]. In addition to strong content validity, ASRAP has been shown to have good construct validity and excellent repeatability in patients with SSc [9]. Similarly, prior work has established NFC as vital in SSc-RP patient diagnostic assessments and potentially important for prognosis [18]. Previous studies have suggested that RP symptom characteristics might evolve over time in SSc alongside the progressive obliterative microangiopathy [19]. We hypothesized that SSc-RP microvascular damage as assessed by Dinolite NFC correlates with symptom burden assessed by the ASRAP questionnaire. We found significant associations between ASRAP scores and NFC abnormalities particularly in cold winter months. These data provide mechanistic insight and support the convergent validity of the ASRAP with NFC for measurement of RP in patients with SSc.

Performance of NFC is a cornerstone of SSc diagnostic assessment, and NFC abnormalities have been shown to be related to DU, interstitial lung disease and PAH [20]. Additionally, capillary dropout/loss has been associated with higher mortality in patients with SSc-RP [21]. Despite this, the association between NFC and HRQoL in SSc-RP has not been investigated. Our study demonstrated that dilated capillaries (characteristic of ‘early’ and ‘active’ scleroderma pattern) and capillary dropout (characteristic of the ‘active’ and ‘late’ scleroderma pattern) were significantly associated with elevated ASRAP scores, but microhemorrhages (characteristic of the ‘active’ scleroderma pattern) were not [22]. The median (IQR) RP disease duration of 10 years [5–22] supports the notion that the ASRAP may be particularly useful in assessing patients with longer SSc-RP duration.

Our study found that receiving vasodilatory treatment did not correlate with significant differences in ASRAP. This aligns with several prior studies including a Cochrane review [23] which demonstrated insufficient evidence to support the use of vasodilatory treatment as most studies reported no improvement in SSc-RP quality of life measures from vasodilatory treatment. Potential reasons for this include the possibility that SSc-RP patients who demonstrate abnormal NFC have already incurred vascular damage that may be unresponsive or less responsive to vasodilatory treatment. This further highlights the unmet need to find pharmacotherapeutic agents that address the underlying pathophysiology of RP. Despite prior studies demonstrating associations between NFC changes and PAH [24], our study found no significant associations between the presence of PAH and the ASRAP. This may be a consequence of a small sample size of patients with PAH in our study but may also indicate that RP symptom burden depends on several factors including vascular disease as well as the neurohumoral axis.

The severity of RP is closely related to seasonal variation [25]. We identified a moderate correlation between the NFC semi-quantitative and ASRAP scores with stronger correlation over cold weather months. The lack of a strong association may stem from the complex pathogenesis of RP that is thought to involve increased microvascular reactivity, along with oxidative stress, endothelial dysfunction and neurovascular dysregulation favouring vasoconstriction [26]. While NFC quantifies microvascular damage, additional tools may be needed to measure impact of other pathogenic mechanisms on RP [27]. Future longitudinal studies involving NFC and ASRAP measurement for the same patients in different seasons are warranted to elucidate this association.

Strengths of the study design included consecutive and prospective recruitment in a real-world academic rheumatology clinic setting and NFC measurement by two assessors blinded to other patient data. Our results fill a gap in the literature by demonstrating significant associations between NFC and RP symptom burden and support the construct validity of the ASRAP for SSc-RP assessment. Our findings have implications for cohort enrichment to SSc-RP clinical trials which traditionally target patients with more severe RP symptoms; however, these patients may also have advanced capillary dropout which may diminish their ability to show improvement to vasodilators. Study weaknesses include the relatively small sample size, our cross-sectional study design and low numbers of Hispanic, black and male patients that limit generalizability. Future large, multicentre and longitudinal research studies should be designed to investigate the prognostic value of the ASRAP and NFC in SSc-RP. The integration of ASRAP and Dinolite NFC to clinical and research settings has the potential to enhance patient care in SSc-RP by advancing our understanding of this complex condition.

## Supplementary Material

rkaf122_Supplementary_Data

## Data Availability

The data underlying this article will be shared on reasonable request to the corresponding author.
