# Supplementary material for: Insight from convergent validity of assessment of systemic sclerosis–associated Raynaud phenomenon with nailfold capillaroscopy
Source: Rheumatol Adv Pract. 2025 Oct 21;9(4):rkaf122. doi: 10.1093/rap/rkaf122 (PMC12574970; doi:10.1093/rap/rkaf122)
Supplement: rkaf122_Supplementary_Data [file rkaf122_supplementary_data.zip › 25-097 Supplementary material.docx]

Supplementary material

| **Supplementary Table S1** - Definitions of specific NFC abnormalities | |
| --- | --- |
| Tortuous capillary | Capillary limb curled but not crossing over |
| Dilated capillary | Width more than two times surrounding capillaries |
| Meandering capillary | Limbs crossed upon themselves |
| Irregularly enlarged capillary | Width of capillary greater than ten times normal |
| Neoangiogenesis/ bushy capillary | Small multiple buds originating from distal loop |
| Microhemorrhages | Micropetechiae |
| Capillary drop out | Absence of individual capillary loop |
| Avascular area | Absence of two or more adjacent capillary loops |
| Bizarre capillaries | Atypical morphology |

| **Supplementary Table S2** – Univariate Regression Model Representing Association Between ASRAP Score and Patient/Disease Characteristics | |
| --- | --- |
| Parameter | β (Standard Error) |
| **Age (per 1 year)** | -0.07 (0.09) |
| **Sex (Male vs female)** | 11.99 (3.32) * |
| **Race/Ethnicity (ref: white, not Hispanic or Latino)** |  |
| *Black or African American* | 3.85 (4.19) |
| *Hispanic or Latino* | 11.25 (3.91) * |
| *Other* | -4.48 (9.87) |
| **SSc Disease Subtype (ref: IcSSc)** |  |
| *DcSSc* | -0.58 (3.42) |
| *SSc Sine* | -8.54 (4.85) |
| *MCTD* | 13.99 (10.49) |
| **Anti-Centromere Antibodies** |  |
| *Negative* | -0.719 (-2.48) |
| **Anti Scl-70 Antibodies** |  |
| *Negative* | -1.30 (3.17) |
| **Anti RNA Polymerase III Antibodies** |  |
| *Negative* | -1.45 (4.00) |
| **Modified Rodnan Skin Score** | 0.10 (0.20) |
| **History of digital ulcers (ref: YES)** | -7.90 (3.21) * |
| **Diagnosis of pulmonary hypertension (ref: YES)** | 1.331 (3.603) |
|  |  |
| **Mean Semi Quantitative Score (ref: No Changes)** |  |
| **≤ 33% of Capillary Alterations/Reduction** | 7.213 (3.61) * |
| **34–66% of Capillary Alterations/Reduction** | 1.338 (3.81) |
| **≥ 67% of Capillary Alterations/Reduction** | 9.716 (3.48) * |
| **Receiving treatment for RP (ref: YES)** | -0.25 (2.53) |
| **p<0.05* | |

| **Supplementary Table S3** – Multivariate Regression Model Representing Association Between Specific Abnormality Presence and ASRAP Score Controlling for Specific Patient Characteristics | |
| --- | --- |
| Parameter | β (Standard Error) |
| **Age (per year)** | -0.03 (0.08) |
| **Sex (Male vs female)** | 11.18 (3.30) * |
| **Race/Ethnicity (ref: white, not Hispanic or Latino)** |  |
| - *Black or African American* | 0.56 (4.49) |
| - *Hispanic or Latino* | 4.81 (3.93) |
| - *Other* | -5.81 (4.74) |
| ***History of Digital Ulcers (ref: YES)*** | -2.34 (3.35) |
| **Season of Measurement (ref: WINTER)** |  |
| Fall | -4.10 (2.58) |
| - Spring | 2.60 (3.26) |
| - Summer | -3.24 (3.73) |
| - **Mean Semi Quantitative Score (ref: No Changes)** |  |
| **≤ 33% of Capillary Alterations/Reduction** | 4.53 (3.37) |
| **34–66% of Capillary Alterations/Reduction** | -0.18 (4.01) |
| **≥ 67% of Capillary Alterations/Reduction** | 3.00 (4.80) |
| **Tortuous capillary** | 3.72 (3.91) |
| **Dilated capillary** | 6.10 (2.87) * |
| **Meandering capillary** | -4.34 (2.82) |
| **Neoangiogenesis** | 2.41 (3.25) |
| **Giant capillary** | -0.90 (4.03) |
| **Microhemorrhages** | -3.71 (2.83) |
| **Capillary drop out** | 6.84 (3.29) * |
| **Avascular area** | -3.33 (2.54) |
| **Bizarre capillaries** | 0.73 (2.42) |
| **p<0.05, significant result* |  |
|  | |


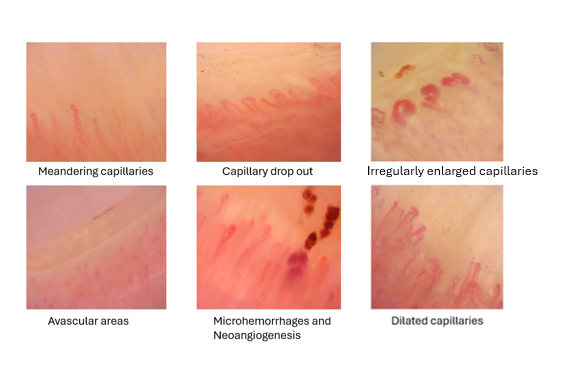


**Supplementary Figure S1:** **Images of specific nailfold capillary abnormalities.**

Alt text: Representative images of specific nailfold capillary abnormalities captured using Dinolite under 200X magnification.
